# Retraction: The expression of ephrinA1/ephA2 receptor increases in chronic rhinosinusitis and ephrinA1/ephA2 signaling affects rhinovirus-induced innate immunity in human sinonasal epithelial cells

**DOI:** 10.3389/fimmu.2024.1456378

**Published:** 2024-07-05

**Authors:** Frontiers Editorial Office

Please find the retraction statement below:

Following publication, concerns were raised regarding the integrity of the images in the published figures. The authors failed to provide a satisfactory explanation during the investigation, which was conducted in accordance with Frontiers’ policies.

This retraction was approved by the Chief Editors of Frontiers in Immunology and the Editor-in-Chief of Frontiers. The authors have not responded to correspondence regarding this retraction.

